# RhoA and Rac1 GTPases Differentially Regulate Agonist-Receptor Mediated Reactive Oxygen Species Generation in Platelets

**DOI:** 10.1371/journal.pone.0163227

**Published:** 2016-09-28

**Authors:** Huzoor Akbar, Xin Duan, Saima Saleem, Ashley K. Davis, Yi Zheng

**Affiliations:** 1 Department of Biomedical Sciences, Heritage College of Osteopathic Medicine, Ohio University, Athens, OH, 45701, United States of America; 2 Division of Experimental Hematology and Cancer Biology, Children’s Hospital Medical Center, University of Cincinnati, Cincinnati, OH, 45229, United States of America; University of Kentucky, UNITED STATES

## Abstract

Agonist induced generation of reactive oxygen species (ROS) by NADPH oxidases (NOX) enhances platelet aggregation and hence the risk of thrombosis. RhoA and Rac1 GTPases are involved in ROS generation by NOX in a variety of cells, but their roles in platelet ROS production remain unclear. In this study we used platelets from RhoA and Rac1 conditional knockout mice as well as human platelets treated with Rhosin and NSC23767, rationally designed small molecule inhibitors of RhoA and Rac GTPases, respectively, to better define the contributions of RhoA and Rac1 signaling to ROS generation and platelet activation. Treatment of platelets with Rhosin inhibited: (a) U46619 induced activation of RhoA; (b) phosphorylation of p47^phox^, a critical component of NOX; (c) U46619 or thrombin induced ROS generation; (d) phosphorylation of myosin light chain (MLC); (e) platelet shape change; (f) platelet spreading on immobilized fibrinogen; and (g) release of P-selectin, secretion of ATP and aggregation. Conditional deletion of *RhoA* or *Rac1* gene inhibited thrombin induced ROS generation in platelets. Addition of Y27632, a RhoA inhibitor, NSC23766 or Phox-I, an inhibitor of Rac1-p67^phox^ interaction, to human platelets blocked thrombin induced ROS generation. These data suggest that: (a) RhoA/ROCK/p47^phox^ signaling axis promotes ROS production that, at least in part, contributes to platelet activation in conjunction with or independent of the RhoA/ROCK mediated phosphorylation of MLC; and (b) RhoA and Rac1 differentially regulate ROS generation by inhibiting phosphorylation of p47^phox^ and Rac1-p67^phox^ interaction, respectively.

## Introduction

Blood platelets play a critical role in atherothrombosis [1]. Following a rupture of an atherosclerotic plaque or a vascular injury platelets come in contact with sub-endothelial extracellular matrix and undergo rapid activation that results in cytoskeletal reorganization, platelet shape change, granular secretion, aggregation and thrombus formation. Rho GTPases, including RhoA, Rac1, Cdc42, and RhoG, belonging to the Ras-related small molecular G proteins, have been shown to regulate platelet lamellipodia [2–4] and filopodia [5] formation, platelet spreading [6], retraction [7], secretion [5, 8–12] and aggregation [5, 8–10, 13].

Agonist induced generation of reactive oxygen species (ROS) including superoxide anion (O^-^_2_) and hydrogen peroxide (H_2_O_2_) enhance platelet aggregation and hence the risk of thrombosis [14, 15]. Although diverse biochemical reactions contribute to ROS generation, NADPH oxidases (NOX) have emerged as critical sources of agonist induced ROS generation [16]. Two isoforms of NOX, namely NOX1 and NOX2 and their regulatory subunits p22^phox^, p47^phox^ and p67^phox^, have been characterized in platelets [14, 15, 17, 18] and recent reports have confirmed that ROS activities play a role in regulation of platelet activation [18–21]. In particular, Delaney *et al*. [18] have recently reported that ROS generation by NOX leads to platelet secretion and aggregation via the Syk/phospholipase Cγ2/calcium signaling pathway.

Previous studies have shown that small GTPases RhoA [22] and Rac1 [23, 24] are involved in NOX activation. RhoA can trigger ROS generation via the ROCK mediated phosphorylation of p47^phox^ [22] whereas Rac GTPases activate NOX by interacting with p67^phox^ to promote its binding to NOX [16, 24]. Moreover, it has been shown that ROS generation by Rac1 does not involve phosphorylation of p47^phox^ [24].

Agonist-receptor induced phosphorylation of myosin light chain (MLC) via Gα_13_/RhoA/ROCK leads to platelet shape change and secretion [25]. However, RhoA is activated not only by agonist-receptor mediated activation of Gα_13_, but also directly and reversibly by ROS leading to stress fiber formation [26]. The bidirectional positive feedback loops for activation of RhoA by ROS and generation of ROS by RhoA suggest that a RhoA-ROS signaling circuit is involved in regulation of platelet activation. Reports that inhibition of RhoA blocks activation of ROCK as well as phosphorylation of p47^phox^ and ROS production [22] further support the possibility that a RhoA/ROS signaling contributes to platelet activation. In this study we investigated the effects of gene targeting or pharmacologic inhibition of RhoA on ROS generation by thrombin or U46619, two of the agonists known to activate RhoA [27] and generate ROS [20], to better understand the role of RhoA in ROS generation and platelet activation.

We have shown earlier that deficiency or inhibition of Rac1 GTPase inhibits platelet secretion and aggregation induced by diverse agonists including thrombin and U46619 [8]. Although Rac1 GTPase has been shown to be critical in ROS generation by NOX enzymes [16] so far its role in agonist induced ROS generation in platelets has remained to be determined.

Here we report that RhoA, through ROCK/p47^phox^ signaling, generates ROS that, at least in part, contributes to platelet activation in conjunction with or independent of the RhoA mediated ROCK phosphorylation of MLC. Our data show that RhoA and Rac1 differentially regulate ROS generation by inhibiting phosphorylation of p47^phox^ and Rac1-p67^phox^ interaction, respectively.

## Materials and Methods

### Materials

Rhosin was custom synthesized as described [28]. Collagen was obtained from Chrono-Log Corporation (Havertown, PA). The anti-PAK, anti-phospho-PAK, anti- p47phox and anti-GAPDH antibodies were purchased from Cell Signaling Technology, Boston, MA. The anti- phosho-p47phox was purchased from MyBioSource, San Diego, CA. HRP-conjugated goat anti-mouse IgG and HRP-conjugated goat anti-rabbit IgG were obtained from Thermo Scientific–Pierce, Rockford, IL. All other chemicals and reagents were purchased either from Sigma-Aldrich or from specifically noted sources.

### Methods

#### RhoA and Rac1 knockout mice

Conditional RhoA or Rac1 knockout mice, Mx-Cre;RhoA^loxP/loxP^, Mx-Cre;Rac1^loxP/loxP^, inducible deletion of RhoA or Rac1gene by poly I:C induction, and blood platelet harvest, were described previously [29, 30]. All animal maintenance and procedures were approved by Cincinnati Children’s Institution Animal Care and Utility Committee (Protocol # 1E05054).

#### Collection of blood and preparation of washed platelet suspensions

All experiments using human blood from healthy volunteers were performed according to the protocols approved by the Institutional Review Board at Ohio University (Protocol # 08X126), Athens, Ohio or Cincinnati Children’s Hospital Research Foundation (Protocol # 2010–1855), Cincinnati, Ohio. Each volunteer was required to sign an informed consent form approved by the appropriate Institutional Review Board. Procedures for drawing human blood, isolation of platelet-rich plasma (PRP) and preparation of washed platelet suspensions are the same as reported earlier [8, 31]. The platelet count was adjusted to 3 x 10^8^ per ml for aggregation studies.

#### RhoA, Rac1 and Cdc42 GTPase Assays

The relative levels of RhoA-GTP, Cdc42-GTP and Rac1-GTP in washed human platelets were quantified by the effector domains of GST-Rhotekin or GST-PAK1 pull down assays as reported earlier [28, 32]. The GTP-bound RhoA, Cdc42 or Rac1 were quantitatively detected by Western blotting using anti-RhoA, anti-Cdc42 (Cell Signaling Technology, Boston, MA) and anti-Rac1 (BD Transduction, San Jose, CA) antibodies respectively.

#### ROS generation

Washed platelets were incubated with 2’7’-dichlorofluorescein (dcf-da10 μM) for 15 minutes at 37°C, washed once more to remove extracellular dye and ROS was detected by flow cytometry. ROS generation is expressed as a % of ROS in stimulated platelets. The mean fluorescence intensity or the mean percentage of dcf-positive platelets were used to calculate ROS generation.

#### Phosphorylation of MLC and p47^phox^

Washed human platelets were stimulated with U46619 or thrombin for a specified time period. The reactions were terminated by addition of 5x sample buffer and phosphorylated proteins were detected by Western blotting as described earlier [8].

#### Platelet shape change, release of P-selectin, ATP secretion and platelet aggregation

Platelet shape change was monitored in washed platelets using an Aggregometer. The decrease in the light transmittance following addition of an agonist represents platelet shape change. P-selectin release from the α-granules was quantified by flow cytometry as described earlier [5]. Secretion of ATP from the dense granules was assessed by a luminescence method using a luciferin/luciferase kit and a Lumi-Aggregometer from Chrono-Log Corporation (Havertown, PA) [5]. Platelet aggregation was monitored as reported earlier using a Lumi-Aggregometer at 37°C and a stirring speed of 900 rpm [5].

#### Assessment of platelet spreading on immobilized fibrinogen

Glass cover slips were coated with fibrinogen overnight at 4°C. Non-specific binding was blocked by incubating cover slips with bovine serum albumin (BSA, 1%) in Tyrode’s-HEPES buffer at 37°C. Cover slips were rinsed with Tyrode’s-HEPES buffer after removing BSA. Aspirin (1mM) treated washed murine platelets containing apyrase (3 U/ml) were layered over cover slips in the presence or absence of Rhosin. After a five minute incubation at 37°C the cover slips were rinsed with PBS to remove free platelets. Platelets on cover slips were then fixed with 4% paraformaldehyde for ten minutes, rinsed with PBS twice and permeabilized with 0.1% Triton X-100 for 60 seconds. After two rinses with PBS platelets were stained with Alexa 594-phalloidin to visualize F-actin [5]. A Carl Zeiss LSM-510 confocal Axioplan 200 microscope and a Plan-Neofluar 100x/1.45 oil objective was used to generate platelet images. Digital images were processed using Zen 2007 software from Carl Zeiss.

#### Statistical analysis

Data are expressed as means ± SD or SE as described in figure legend. A *p* value of <0.05 indicates statistically significant difference between the control and test samples.

## Results

### Rhosin inhibited RhoA GTPase activation in platelets

**Fig 1:**
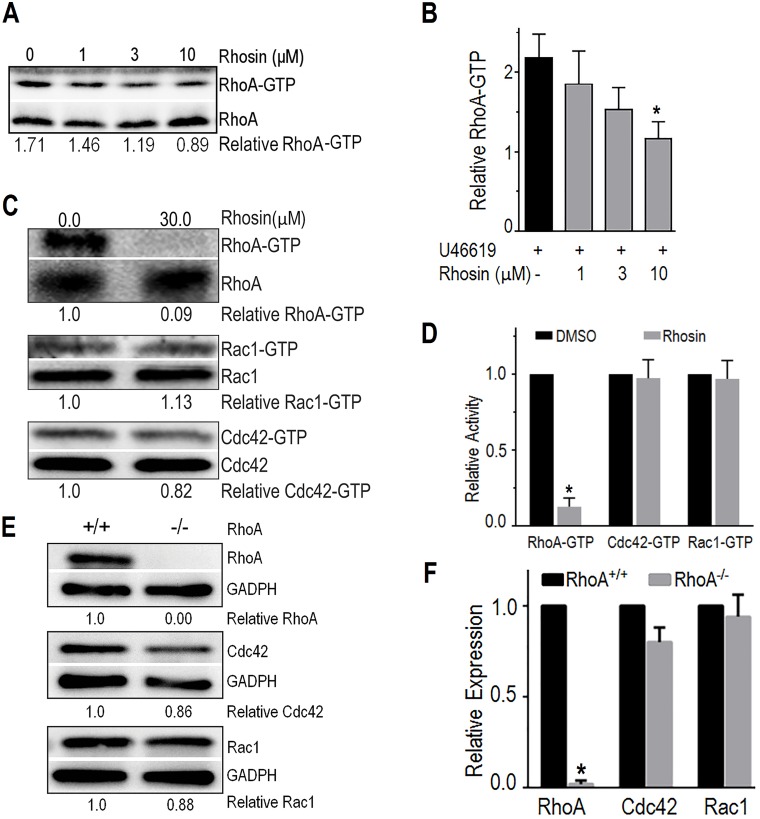
Rhosin inhibited RhoA GTPase activation and gene targeting of RhoA deleted expression of RhoA in platelets. **(A-D)** Washed human platelets were incubated with U46619 (0.01 μM) for one minute. The reactions were terminated by adding ice-cold HEPES-buffered Tyrode’s solution containing protease inhibitors cocktail. GTP loading of RhoA, Rac1 and Cdc42 was analyzed as described in the methods section. A two minute pre-incubation of platelets with Rhosin inhibited U46619 induced RhoA-GTP formation in a concentration-dependent manner. Rhosin minimally inhibited Cdc42-GTP and Rac1-GTP formation. **(E-F)** Conditional RhoA knockout mice were generated as described previously [29]. The Western blots and the bar graph show that gene targeting of RhoA completely deleted RhoA expression and partially decreased Cdc42 or Rac1 expression in platelets. Total RhoA, Rac1, Cdc42 and GADPH are shown as loading controls. The data in bar graphs are mean ± SE from three experiments (*p<0.05).

We have shown earlier that Rhosin specifically inhibits activation of RhoA in the NIH 3T3 cells by binding to RhoA at the site required for its activation by Rho-GEF [28]. In this study we investigated the effects of Rhosin on activation of platelet Rho GTPases to demonstrate that Rhosin specifically inhibits activation of RhoA but not that of Rac1 and Cdc42. Washed human platelets were incubated with Rhosin or DMSO for two minutes and then stimulated withTXA_2_ analog U46619, a known inducer of RhoA activation. Blots in Fig 1A and 1C show that Rhosin inhibited U46619 induced RhoA activation in a concentration-dependent manner. The data in bar graph (Fig 1D) show that Rhosin significantly inhibited RhoA activation with minimal effects on Rac1 or Cdc42 activation.

### Gene targeting of RhoA deleted expression of RhoA in platelets

RhoA induces ROS generation via ROCK mediated phosphorylation of p47phox [22]. We investigated the possibility that if RhoA is involved in ROS generation then genetic deficiency of RhoA should inhibit ROS generation. To test this possibility we generated RhoA deficient mice as detailed in the methods section and characterized expression of RhoA and related Rho proteins Rac1 and Cdc42 in washed platelets from *RhoA*^*-/-*^ and matching *RhoA*^*+/+*^ mice by Western blotting. Blots in Fig 1E and the bar graph (Fig 1F) show that gene targeting of *RhoA* completely depleted RhoA with a minimal effect on the expression of Rac1 or Cdc42.

### Inhibition of RhoA GTPase blocked phosphorylation of p47^phox^

**Fig 2:**
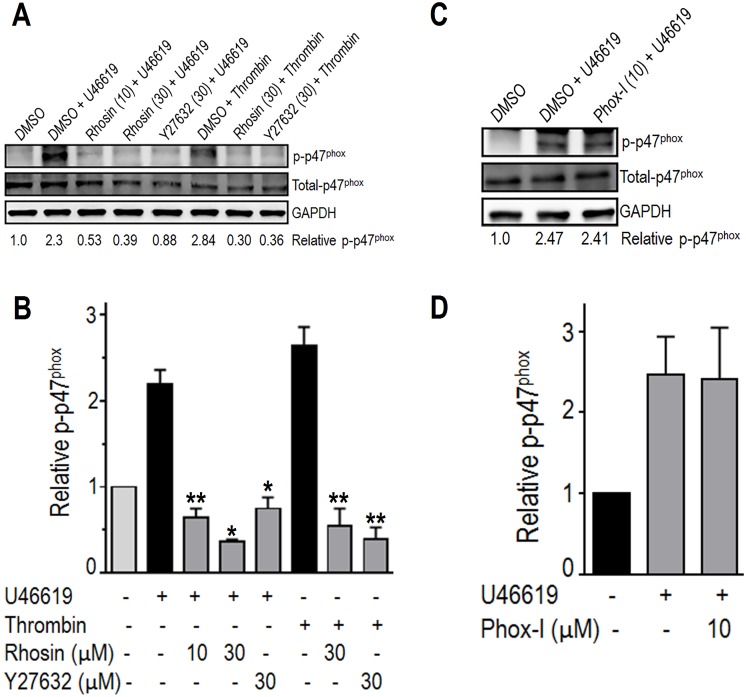
Rhosin and Y27632 but not Phox-I inhibited phosphorylation of p47^phox^. (**A-B**) A two minute incubation of washed human platelets with U46619 (0.5 μM) induced phosphorylation of p47^phox^. Addition of Rhosin (10, 30 μM) or Y27632 (30 M), a known inhibitor of RhoA, two minutes prior to stimulation with U46619 or thrombin inhibited phosphorylation of p47^phox^. **(C-D)** A two minute pre-incubation of platelets with Phox-I (10 μM), an inhibitor of Rac1-p67^phox^ interaction, did not inhibit phosphorylation of p47^phox^. Phosphorylation of p47^phox^ was quantified by densitometry. Data in the bar graphs are mean ± SE from three experiments (*p<0.01, **p<0.001).

Platelet activation by diverse agonists leads to reactive oxygen species (ROS) generation and ROS have been shown to regulate platelet activation [14]. Based on the reports that RhoA/ROCK mediated phosphorylation of p47^phox^, the organizing subunit of NOX, regulates ROS generation and ROS in turn directly activates RhoA, we investigated the possibility that RhoA also regulates platelet activation by affecting ROS generation. A two minute pre-incubation of washed human platelets with Rhosin inhibited U46619 or thrombin induced phosphorylation of p47^phox^ (Fig 2A). Addition of Y27632, a known inhibitor of RhoA/ROCK signaling [27], to platelets also blocked phosphorylation of p47^phox^ (Fig 2A). However, Phox-I, an inhibitor of Rac1-p67phox interaction [33], necessary for NOX mediated ROS generation, did not inhibit phosphorylation of p47phox (Fig 2C). Quantitative analysis of these blots indicates that inhibition of RhoA GTPase by Rhosin or Y27632 prevents phosphorylation of p47^phox^ (Fig 2B) whereas inhibition of Rac1-p67phox interaction does not affect phosphorylation of p47phox (Fig 2D).

### Inhibition of RhoA GTPase blocked ROS generation

**Fig 3:**
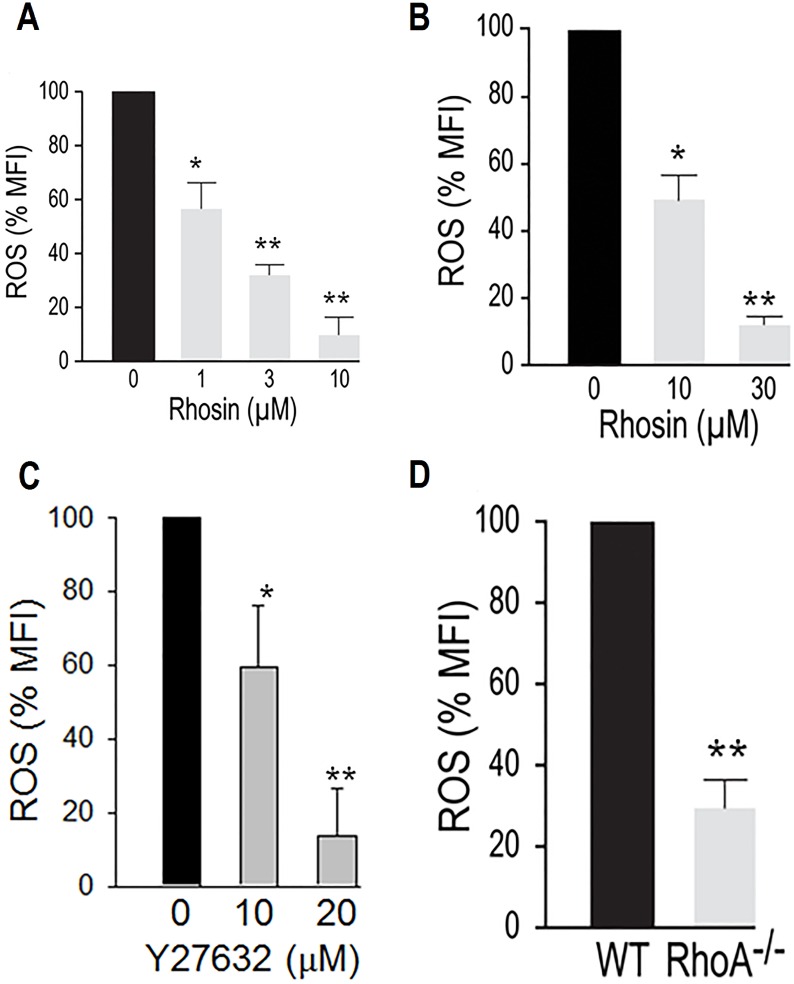
Inhibition of RhoA by Rhosin blocked ROS generation. **(A-B)** Incubation of washed human platelets with Rhosin inhibited U46619 (0.1 μM) or thrombin (0.1 U/ml) induced ROS generation in a concentration-dependent manner. **(C)** Incubation of washed human platelets with Y27632 inhibited thrombin (0.1 U/ml) induced ROS generation. **(D)** Thrombin (0.1 U/ml) induced ROS generation is diminished in RhoA^-/-^, as compared to RhoA^+/+^, platelets. Generation of reactive oxygen species in dcf-da loaded washed platelets was monitored by flow cytometry as detailed in the Methods section. (The data are mean ± SE, n = 4. *p<0.01, **p<0.001).

Since activation of p47^phox^ is required for agonist induced ROS generation by NOX, we next investigated the possibility that inhibition of p47phox phosphorylation should prevent ROS generation. Addition of Rhosin to platelets blocked ROS generation induced by U46619 (Fig 3A) or thrombin (Fig 3B). Treatment of platelets with Y27632 also inhibited thrombin induced ROS generation (Fig 3C). We further investigated the role of RhoA in ROS generation using the RhoA^-/-^ platelets. As shown in Fig 3D, addition of thrombin to platelets from *RhoA*^*-/-*^ mice generated significantly less ROS than platelets from the matching wild type mice. These data indicate that RhoA regulates NOX mediated ROS generation by ROCK mediated phosphorylation of p47^phox^.

### Inhibition of RhoA prevented phosphorylation of myosin light chain and platelet shape change

**Fig 4:**
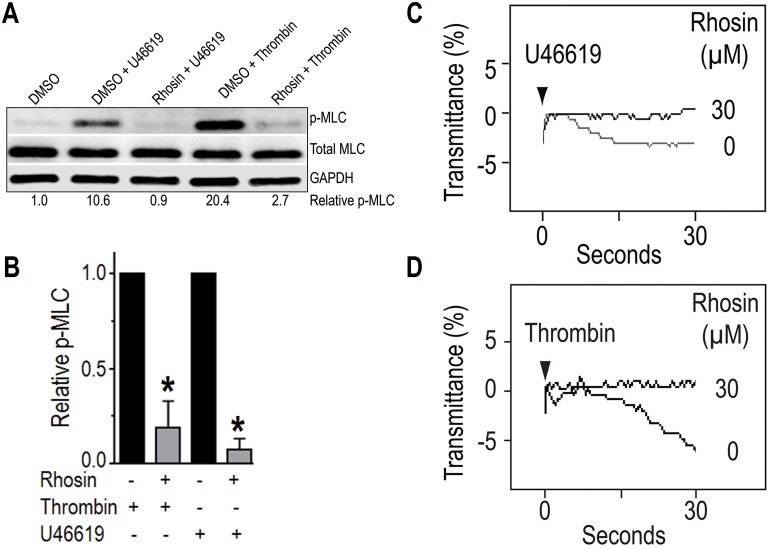
Inhibition of RhoA by Rhosin blocked phosphorylation of myosin light chain and platelet shape change. **(A-B)** Addition of Rhosin (30 μM) to aspirin (1 mM) treated washed human platelets containing apyrase (3 U/ml) two minutes prior to stimulation withU46619 (0.01 μM) or thrombin (0.01 U/ml) blocked phosphorylation of myosin light chain. The reactions were terminated at 30 seconds by adding 5x sample buffer and samples were processed for Western blotting and probed for MLC, p-MLC. Phosphorylation of myosin light chain was quantified by densitometry. Data in the bar graphs are mean ± SE from three experiments (*p<0.001). **(C-D)** Aspirin (1 mM) treated washed human platelets containing apyrase (3 U/ml) were incubated with U46619 (0.01 μM) or thrombin (0.005 U/ml) and platelet shape change was recorded as a decrease in light transmittance using a Lumi-Aggregometer. A two minute pre-incubation with Rhosin blocked platelet shape change by U46619 or thrombin. The shape change racings are representative of four independent experiments.

Activated RhoA increases phosphorylation of myosin light chain (MLC) via its effector ROCK by inhibiting MLC phosphatase [34]. We investigated the possibility that if Rhosin prevents RhoA activation then it should also inhibit phosphorylation of MLC. Addition of Rhosin (30 μM) to aspirin (1 mM) treated platelets containing apyrase (3 U/ml) two minutes prior to stimulation with U46619 or thrombin inhibited phosphorylation of MLC (Fig 4A and 4B). These findings further confirm that Rhosin is an effective inhibitor of RhoA activation in platelets.

The role of RhoA in inducing phosphorylation of MLC in cytoskeletal reorganization leading to platelet shape change is well known [27]. We investigated the possibility that Rhosin by inhibiting RhoA/ROCK mediated MLC phosphorylation blocks platelet shape change. The effect of Rhosin on platelet shape change was recorded using an aggregometer by monitoring the decrease in light transmittance following addition of an agonist. U46619 or thrombin induced platelet shape change in aspirin (1 mM) treated washed human platelets in the presence of apyrase (3 U/ml). Incubation of platelets with Rhosin (30 μM) inhibited U46619 or thrombin induced platelet shape change (Fig 4C and 4D). These results show that Rhosin blocks U46619 or thrombin induced platelet shape change mediated by RhoA.

### Inhibition of RhoA inhibited platelet spreading on immobilized fibrinogen

**Fig 5:**
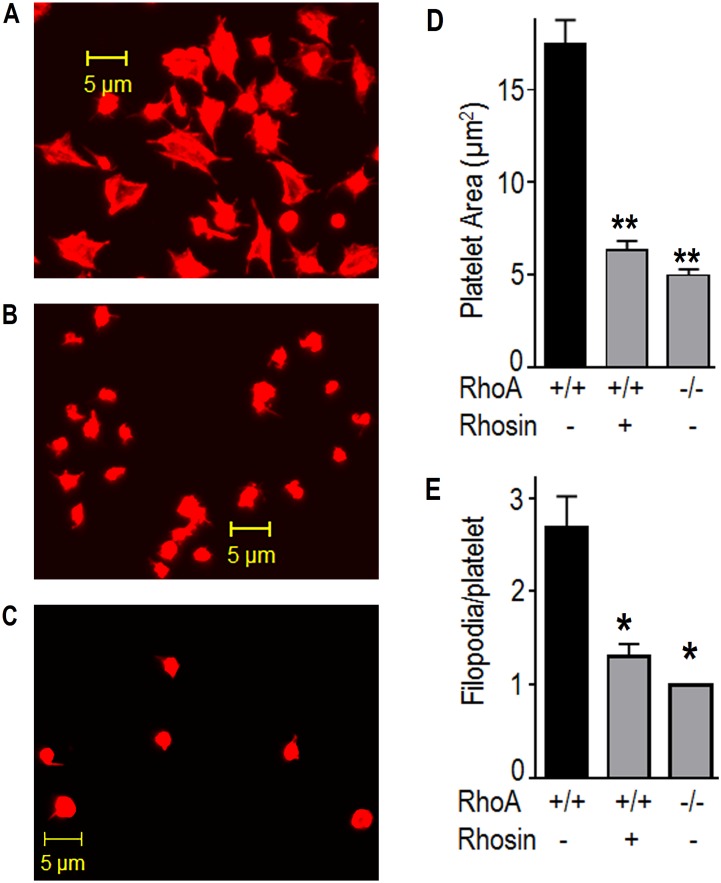
Inhibition of RhoA by Rhosin or gene targeting of RhoA diminished platelet spreading on immobilized fibrinogen. **(A-C)** RhoA^+/+^ platelets (A), RhoA^+/+^ platelets with Rhosin (30 μM, B) or RhoA^-/-^ platelets (C) were layered over fibrinogen (3 μg/ml) coated cover slips in the presence of apyrase (3 U/ml) for five min. The cover slips were washed and adherent platelets were processed for immuno-fluorescence confocal microscopy as detailed in the methods section. Platelets treated with Rhosin (B) and RhoA^-/-^ platelets (C), as compared to DMSO (A) exhibited diminished spreading and filopodia formation on immobilized fibrinogen. **(D-E)** The bar graphs show that spreading of Rhosin treated (n = 23) or RhoA-deficient (n = 23, S4 Fig), as compared to the matching RhoA^+/+^ platelets (n = 28) was diminished significantly (**p<0.001). The Rhosin treated or RhoA-deficient platelets exhibited a significant decrease in the number of filopodia (*p<0.01). Spreading of washed platelets on fibrinogen was quantified using Image J software (http://rsbweb.nih.gov/ij).

Binding of ligands such as fibrinogen to integrin αIIbβ_3_ induces outside-in signaling leading to cytoskeletal reorganization that results in morphological changes namely platelet spreading, formation of filopodia, lamellipodia and stress fibers. RhoA has been shown to be involved in platelet cytoskeletal reorganization. We therefore investigated the possibility that inhibition of RhoA by Rhosin may prevent outside-in signaling mediated platelet spreading. Aspirin (1mM) treated washed RhoA^+/+^ platelets with or without Rhosin and RhoA^-/-^ platelets were layered over immobilized fibrinogen and platelet morphological changes were visualized by confocal microscopy. Rhosin treated platelets (Fig 5B), and RhoA-deficient platelets (Fig 5C), as compared to matching controls (Fig 5A) exhibited significantly less spreading on fibrinogen and a decrease in the numbers of filopodia. Platelet spreading was diminished in Rhosin treated or RhoA^-/-^ platelets by 64% and 71% respectively (Fig 5D). Eighty percent of the DMSO treated, 44% of Rhosin treated and only 22% of RhoA^-/-^ platelets exhibited filopodia. In platelets expressing filopodia Rhosin or RhoA deficiency decreased the number of filopodia by 51% and 63% respectively (Fig 5E). These findings clearly show that RhoA plays a critical role in integrin mediated cytoskeletal reorganization and its deficiency due to gene targeting or inhibition by Rhosin diminishes platelet spreading.

### Inhibition of RhoA blocked platelet release of p-selectin, ATP secretion and aggregation

**Fig 6:**
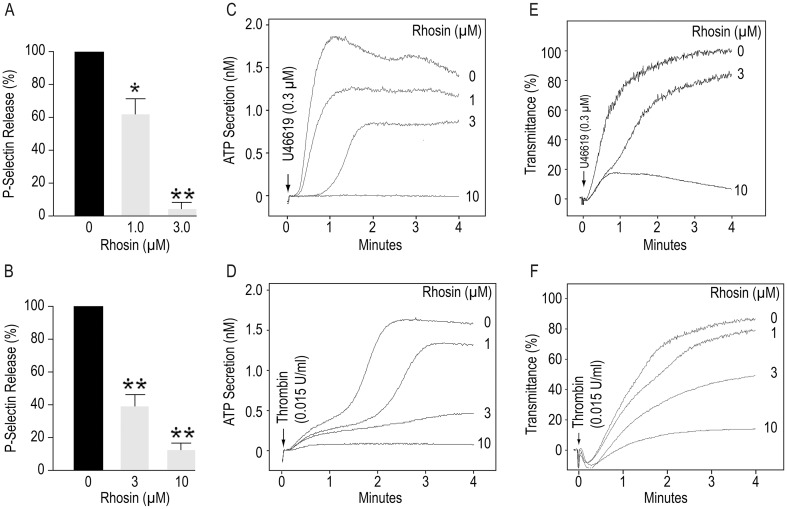
Inhibition of RhoA by Rhosin blocked U46619 or thrombin induced release of P-selectin, secretion of ATP and platelet aggregation. **(A-B)** Incubation of washed human platelets with U46619 or thrombin induced release of p-selectin from platelet α-granules. Addition of Rhosin to platelets two minutes prior to stimulation with U46619 or thrombin inhibited expression of p-selectin in a concentration dependent manner. P-selectin was quantified by flow cytometry in aspirin treated (1 mM) washed platelets, containing 0.2% bovine serum albumin and apyrase (0.4 U/ml) as detailed in the methods section. Results are reported as means ± SD (n = 4, *p<0.01, **p<0.001). **(C-D)** Addition of U46619 or thrombin to washed human platelets induced ATP secretion and **(E-F)** platelet aggregation. A two minute pre-incubation with Rhosin inhibited ATP secretion and platelet aggregation in a concentration dependent manner. A Lumi-Aggregometer from Chrono-Log-Corporation (Havertown, PA) was used to monitor platelet ATP secretion and aggregation. The secretion and aggregation tracings are representative of 3 independent experiments.

Pharmacologic inhibition or gene targeting of RhoA has been shown to result in defective platelet function [35–37]. RhoA deficiency has been reported to result in significantly diminished release of P-selectin in response to thrombin and impaired aggregation induced by thrombin or protease activated receptor peptide-4 [35]. We investigated the possibility that if RhoA is involved in platelet secretion and aggregation than inhibition of RhoA by Rhosin should block secretion from the dense and α-granules as well as platelet aggregation. Addition of Rhosin to platelets two minutes before stimulation with U46619 or thrombin inhibited release of P-selectin (Fig 6A and 6B), secretion of ATP (Fig 6C and 6D) and aggregation (Fig 6E and 6F) in a concentration-dependent manner. These data indicate that RhoA plays a critical role in platelet activation.

### Inhibition or gene targeting of Rac1 GTPase blocked ROS generation

**Fig 7:**
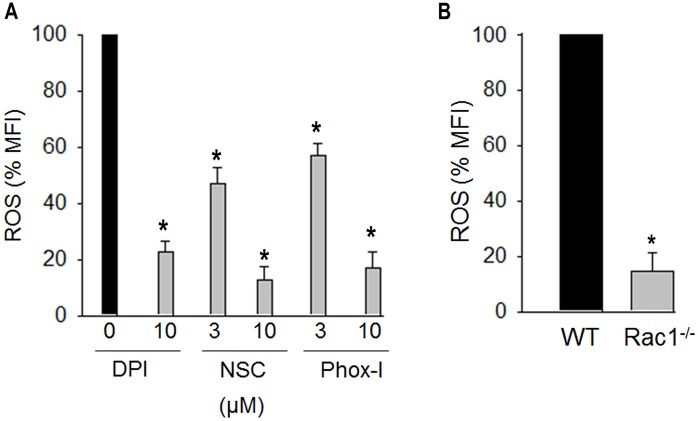
Deficiency or inhibition of Rac1 GTPase or inhibition of Rac1-P67^phox^ interaction prevents ROS generation. **(A)** Incubation of washed human platelets with NSC23766 or Phox-I inhibited thrombin (0.1 U/ml, mean ± SE, n = 4, *p < 0.001) induced ROS generation. Diphenyleneiodonium (DPI), a non-selective inhibitor of ROS generation also inhibited thrombin induced ROS generation. **(B)** Conditional Rac1 knockout mice were generated as described previously [30]. Addition of thrombin (0.1 U/ml) induced significantly less ROS generation in Rac1^-/-^, as compared to Rac1^+/+^, platelets (mean ± SE, n = 4, *p < 0.001). Generation of reactive oxygen species in dcf-da loaded washed platelets was monitored by flow cytometry as detailed in the Methods section.

The role of Rac GTPases in ROS generation by NOX1 and NOX2 has been well documented [16]. Rac GTPases activate NOX by enhancing binding of p67^phox^ to NOX2 [16]. If Rac1 GTPase is essential for ROS generation then gene targeting or inhibition of Rac GTPases should inhibit ROS generation in platelets. To test this possibility we investigated ROS generation in platelets from *Rac1*^*-/-*^ mice and platelets treated with NSC23766, a specific inhibitor of Rac GTPases [38]. Addition of NSC23766 to washed human platelets two minutes before stimulation with thrombin blocked ROS generation (Fig 7A). As shown in Fig 7B, thrombin stimulation led to significantly less ROS production in platelets from Rac1^-/-^ mice as compared with platelets from the matching wild type mice. These data indicate that Rac1 GTPase plays a critical role in agonist induced ROS generation in platelets.

Interaction of activated i.e. GTP bound Rac GTPases with p67^phox^ is essential for activation of NOX leading to ROS generation [16, 39–41]. We investigated the effects of Phox-I, a rationally designed small molecule inhibitor of Rac1-P67^phox^ interaction [33] to determine if inhibition of Rac1-p67^phox^ interaction prevents ROS generation. Addition of Phox-I to platelets two minutes before stimulation with thrombin inhibited ROS generation in platelets (Fig 7A). These data suggest that the regulation of ROS generation by Rac1 depends on Rac1-p67^phox^ interaction.

## Discussion

A possible role for ROS in platelet activation was reported more than thirty-nine years ago [42, 43]. Since then ROS generation in platelets stimulated by diverse agonists has been shown by flow cytometry [20] and a number of reports have shown that agonist induced ROS generation in platelets involves NADPH oxidase (NOX) [20, 44–46]. Platelets have been shown to express NOX1 and NOX2 [17, 18] and patients with an inherited deficiency of NOX2 also known as gp91^phox^ have been reported to have impaired platelet function [47]. Rho family GTPases Rac1 and RhoA have been shown to regulate ROS generation by NOX. RhoA has been shown to play a critical role in agonist-receptor mediated platelet activation via the classical RhoA/ROCK/MLC phosphorylation pathway [35–37, 48]. However, platelet activation by diverse agonists not only leads to phosphorylation of MLC but also phosphorylation of p47^phox^ and ROS generation. The ability of ROS to directly and reversibly activate RhoA leading to stress fiber formation [26] suggests that activation of RhoA is yet another mechanism involved in ROS mediated platelet activation. In this study thrombin and U46619, two of the agonists known to activate RhoA GTPase, were used to better understand the role of RhoA in ROS generation and platelet activation.

Our findings that platelets treated with Rhosin, a rationally designed small molecule inhibitor of RhoA [28], and platelets from RhoA^-/-^ mice generated less ROS in response to thrombin (Fig 3B and 3D) shows for the first time that RhoA plays a critical role in agonist-induced ROS generation in platelets. Inhibition of thrombin induced ROS generation in platelets treated with Y27632 (Fig 3C), a known inhibitor of RhoA [27], or in platelets from *RhoA*^*-/-*^, as compared to *RhoA*^*+/+*^, platelets (Fig 3D) provide further evidence that RhoA is involved in agonist induced ROS generation in platelets.

A role of agonist/RhoA/ROCK mediated MLC phosphorylation in platelet shape change and secretion has been known for some time [36, 37, 48]. Inhibition of RhoA blocks downstream effector ROCK and consequently ROCK mediated phosphorylation of target proteins including MLC. Our findings that Rhosin inhibited thrombin or U46619 induced phosphorylation of MLC (Fig 4A and 4B), and shape change (Fig 4C and 4D) in the presence of aspirin and apyrase, inhibitors of secondary mediators namely TXA_2_ and ADP released during platelet activation, suggest that Rhosin inhibits platelet shape change by blocking RhoA/ROCK mediated phosphorylation of MLC. These findings are in agreement with a recent report that gene targeting of *RhoA* abolishes thrombin or U46619 induced phosphorylation of MLC and platelet shape change [35].

Our findings that pharmacologic targeting of RhoA by Rhosin or genetic deletion of RhoA inhibited spreading of platelets on immobilized fibrinogen (Fig 5) suggest that *RhoA* is involved in integrin αIIbβ_3_-dependent spreading of platelets. Others have reported that RhoA is either required or is not needed for platelet spreading to occur [49, 50]. Gong *et al*. [51] have reported that initially integrin αIIbβ_3_-dependent inhibition of RhoA leads to platelet spreading and at the later stage termination of RhoA inhibition leads to RhoA-dependent contraction. Reasons for the discrepancies in the role of RhoA in platelet spreading is not clear at this time. However, different fibrinogen coating densities have been shown to dramatically affect integrin αIIbβ_3_-mediated platelet signaling and spreading [52]. We incubated platelets on fibrinogen (3 μg/ml) for five minutes. Gong *et al*. [51] incubated platelets for 90 minutes on 100 μg/ml fibrinogen coated coverslips [26]. Pleines *et al*. used 200 μg/ml fibrinogen to study spreading of RhoA^-/-^ platelets for thirty minutes [35]. The differences between the density of fibrinogen and or duration of time platelets were exposed to fibrinogen used in our and other studies may be responsible for the discrepancies in our and their observations.

RhoA has been shown to activate NOX by phosphorylating p47phox [22] and NOX generated ROS leads to platelet secretion and aggregation via the Syk/phospholipase Cγ2/calcium signaling pathway [18]. Our findings that Rhosin inhibited RhoA/ROCK/p47^phox^/NOX mediated ROS generation (Fig 3) and U46619 or thrombin induced release of P-selectin, ATP secretion and aggregation (Fig 6) suggests that inhibition of RhoA/ROCK/NOX/ROS, at least in part, prevents platelet activation in conjunction with or independent of the RhoA/ROCK/MLC signaling.

Rac GTPases are integral part of ROS generation by NADPH oxidase isoforms NOX1 and NOX2. Binding of activated Rac GTPases to p67^phox^ activates NOX2 by facilitating binding of p67^phox^ to NOX2. Our findings that inhibition of Rac1 GTPase by NSC23766 (Fig 7A) or gene targeting of Rac1GTPase (Fig 7B) diminished ROS generation in platelets clearly demonstrate that Rac1 is essential for agonist induced ROS generation in platelets. Moreover, our data showing that Phox-I, an inhibitor of Rac1-p67^phox^ interaction, blocks thrombin induced ROS generation (Fig 7A) without affecting phosphorylation of p47^phox^ (Fig 2C and 2D) concur with other published reports [16, 33] that Rac1-p67^phox^/NOX signaling plays a critical role in Rac1 mediated ROS generation. These observations, together with a report by others that Rac1 is not involved in phosphorylation of p47^phox^ [24] suggest that Rac1 and RhoA utilize distinct signaling to regulate agonist induced ROS generation in platelets.

## Supporting Information

S1 Fig Rhosin inhibited RhoA GTPase activation and gene targeting of RhoA deleted expression of RhoA. (TIF)

S2 Fig Phox-I did not inhibit phosphorylation of p47phox. (TIF)

S3 Fig Inhibition of RhoA by Rhosin blocked phosphorylation of myosin light chain. (TIF)

S4 Fig Gene targeting of RhoA diminished platelet spreading on immobilized fibrinogen. (TIF)

## References

[1] Akbar H. Antithrombotic drugs and their complications In: Pathobiology of Human Disease,. McManus LM, Mitchell RN, editors: Elsevier, 10.1016/B978-0-12-386456-7.07914-4; pp. 1613–1627 (2014).

[2] McCarty OJ, Larson MK, Auger JM, Kalia N, Atkinson BT, Pearce AC, et al Rac1 is essential for platelet lamellipodia formation and aggregate stability under flow. The Journal of biological chemistry. 2005;280(47):39474–84. 10.1074/jbc.M504672200 16195235 PMC1395485

[3] Aslan JE, Baker SM, Loren CP, Haley KM, Itakura A, Pang J, et al The PAK system links Rho GTPase signaling to thrombin-mediated platelet activation. American journal of physiology Cell physiology. 2013;305(5):C519–28. 10.1152/ajpcell.00418.2012 23784547 PMC3761148

[4] Aslan JE, Itakura A, Haley KM, Tormoen GW, Loren CP, Baker SM, et al p21 activated kinase signaling coordinates glycoprotein receptor VI-mediated platelet aggregation, lamellipodia formation, and aggregate stability under shear. Arteriosclerosis, thrombosis, and vascular biology. 2013;33(7):1544–51. 10.1161/ATVBAHA.112.301165 . 23640496 PMC3938029

[5] Akbar H, Shang X, Perveen R, Berryman M, Funk K, Johnson JF, et al Gene targeting implicates Cdc42 GTPase in GPVI and non-GPVI mediated platelet filopodia formation, secretion and aggregation. PloS one. 2011;6(7):e22117 10.1371/journal.pone.0022117 21789221 PMC3138762

[6] Shen B, Delaney MK, Du X. Inside-out, outside-in, and inside-outside-in: G protein signaling in integrin-mediated cell adhesion, spreading, and retraction. Current opinion in cell biology. 2012;24(5):600–6. 10.1016/j.ceb.2012.08.011 22980731 PMC3479359

[7] Flevaris P, Li Z, Zhang G, Zheng Y, Liu J, Du X. Two distinct roles of mitogen-activated protein kinases in platelets and a novel Rac1-MAPK-dependent integrin outside-in retractile signaling pathway. Blood. 2009;113(4):893–901. 10.1182/blood-2008-05-155978 18957688 PMC2630274

[8] Akbar H, Kim J, Funk K, Cancelas JA, Shang X, Chen L, et al Genetic and pharmacologic evidence that Rac1 GTPase is involved in regulation of platelet secretion and aggregation. Journal of thrombosis and haemostasis: JTH. 2007;5(8):1747–55. 10.1111/j.1538-7836.2007.02646.x . 17663742

[9] Pandey D, Goyal P, Dwivedi S, Siess W. Unraveling a novel Rac1-mediated signaling pathway that regulates cofilin dephosphorylation and secretion in thrombin-stimulated platelets. Blood. 2009;114(2):415–24. 10.1182/blood-2008-10-183582 . 19429871

[10] Dwivedi S, Pandey D, Khandoga AL, Brandl R, Siess W. Rac1-mediated signaling plays a central role in secretion-dependent platelet aggregation in human blood stimulated by atherosclerotic plaque. Journal of translational medicine. 2010;8:128 10.1186/1479-5876-8-128 21134286 PMC3018435

[11] Goggs R, Harper MT, Pope RJ, Savage JS, Williams CM, Mundell SJ, et al RhoG protein regulates platelet granule secretion and thrombus formation in mice. The Journal of biological chemistry. 2013;288(47):34217–29. 10.1074/jbc.M113.504100 24106270 PMC3837162

[12] Kim S, Dangelmaier C, Bhavanasi D, Meng S, Wang H, Goldfinger LE, et al RhoG protein regulates glycoprotein VI-Fc receptor gamma-chain complex-mediated platelet activation and thrombus formation. The Journal of biological chemistry. 2013;288(47):34230–8. 10.1074/jbc.M113.504928 24106269 PMC3837163

[13] Akbar H, Cancelas J, Williams DA, Zheng J, Zheng Y. Rational design and applications of a Rac GTPase-specific small molecule inhibitor. Methods in enzymology. 2006;406:554–65. 10.1016/S0076-6879(06)06043-5 . 16472687

[14] Krotz F, Sohn HY, Pohl U. Reactive oxygen species: players in the platelet game. Arteriosclerosis, thrombosis, and vascular biology. 2004;24(11):1988–96. 10.1161/01.ATV.0000145574.90840.7d . 15374851

[15] Violi F, Pignatelli P. Platelet NOX, a novel target for anti-thrombotic treatment. Thrombosis and haemostasis. 2014;111(5):817–23. 10.1160/TH13-10-0818 . 24402688

[16] Bedard K, Krause KH. The NOX family of ROS-generating NADPH oxidases: physiology and pathophysiology. Physiological reviews. 2007;87(1):245–313. 10.1152/physrev.00044.2005 . 17237347

[17] Vara D, Campanella M, Pula G. The novel NOX inhibitor 2-acetylphenothiazine impairs collagen-dependent thrombus formation in a GPVI-dependent manner. British journal of pharmacology. 2013;168(1):212–24. 10.1111/j.1476-5381.2012.02130.x 22881838 PMC3570016

[18] Delaney MK, Kim K, Estevez B, Xu Z, Stojanovic-Terpo A, Shen B, et al Differential Roles of the NADPH-Oxidase 1 and 2 in Platelet Activation and Thrombosis. Arteriosclerosis, thrombosis, and vascular biology. 2016;36(5):846–54. 10.1161/ATVBAHA.116.307308 26988594 PMC4850088

[19] Bakdash N, Williams MS. Spatially distinct production of reactive oxygen species regulates platelet activation. Free radical biology & medicine. 2008;45(2):158–66. 10.1016/j.freeradbiomed.2008.03.021 . 18452718

[20] Begonja AJ, Gambaryan S, Geiger J, Aktas B, Pozgajova M, Nieswandt B, et al Platelet NAD(P)H-oxidase-generated ROS production regulates alphaIIbbeta3-integrin activation independent of the NO/cGMP pathway. Blood. 2005;106(8):2757–60. 10.1182/blood-2005-03-1047 . 15976180

[21] Arthur JF, Qiao J, Shen Y, Davis AK, Dunne E, Berndt MC, et al ITAM receptor-mediated generation of reactive oxygen species in human platelets occurs via Syk-dependent and Syk-independent pathways. Journal of thrombosis and haemostasis: JTH. 2012;10(6):1133–41. 10.1111/j.1538-7836.2012.04734.x . 22489915

[22] Kim JS, Kim JG, Jeon CY, Won HY, Moon MY, Seo JY, et al Downstream components of RhoA required for signal pathway of superoxide formation during phagocytosis of serum opsonized zymosans in macrophages. Experimental & molecular medicine. 2005;37(6):575–87. 10.1038/emm.2005.71 . 16391519

[23] Matono R, Miyano K, Kiyohara T, Sumimoto H. Arachidonic acid induces direct interaction of the p67(phox)-Rac complex with the phagocyte oxidase Nox2, leading to superoxide production. The Journal of biological chemistry. 2014;289(36):24874–84. 10.1074/jbc.M114.581785 25056956 PMC4155656

[24] Miyano K, Sumimoto H. Assessment of the role for Rho family GTPases in NADPH oxidase activation. Methods in molecular biology. 2012;827:195–212. 10.1007/978-1-61779-442-1_14 . 22144277

[25] Offermanns S. Activation of platelet function through G protein-coupled receptors. Circulation research. 2006;99(12):1293–304. 10.1161/01.RES.0000251742.71301.16 . 17158345

[26] Aghajanian A, Wittchen ES, Campbell SL, Burridge K. Direct activation of RhoA by reactive oxygen species requires a redox-sensitive motif. PloS one. 2009;4(11):e8045 10.1371/journal.pone.0008045 19956681 PMC2778012

[27] Klages B, Brandt U, Simon MI, Schultz G, Offermanns S. Activation of G12/G13 results in shape change and Rho/Rho-kinase-mediated myosin light chain phosphorylation in mouse platelets. The Journal of cell biology. 1999;144(4):745–54. 10037795 10.1083/jcb.144.4.745 PMC2132941

[28] Shang X, Marchioni F, Sipes N, Evelyn CR, Jerabek-Willemsen M, Duhr S, et al Rational design of small molecule inhibitors targeting RhoA subfamily Rho GTPases. Chemistry & biology. 2012;19(6):699–710. 10.1016/j.chembiol.2012.05.009 22726684 PMC3383629

[29] Zhou X, Florian MC, Arumugam P, Chen X, Cancelas JA, Lang R, et al RhoA GTPase controls cytokinesis and programmed necrosis of hematopoietic progenitors. The Journal of experimental medicine. 2013;210(11):2371–85. 10.1084/jem.20122348 24101377 PMC3804933

[30] Cancelas JA, Lee AW, Prabhakar R, Stringer KF, Zheng Y, Williams DA. Rac GTPases differentially integrate signals regulating hematopoietic stem cell localization. Nature medicine. 2005;11(8):886–91. 10.1038/nm1274 . 16025125

[31] Perveen R, Funk K, Thuma J, Wulf Ridge S, Cao Y, Akkerman JW, et al A novel small molecule 1,2,3,4,6-penta-O-galloyl-alpha-D-glucopyranose mimics the antiplatelet actions of insulin. PloS one. 2011;6(11):e26238 10.1371/journal.pone.0026238 22073153 PMC3206812

[32] Liu W, Feng Y, Shang X, Zheng Y. Rho GTPases in hematopoietic stem/progenitor cell migration. Methods in molecular biology. 2011;750:307–19. 10.1007/978-1-61779-145-1_21 . 21618100

[33] Bosco EE, Kumar S, Marchioni F, Biesiada J, Kordos M, Szczur K, et al Rational design of small molecule inhibitors targeting the Rac GTPase-p67(phox) signaling axis in inflammation. Chemistry & biology. 2012;19(2):228–42. 10.1016/j.chembiol.2011.12.017 22365606 PMC3292765

[34] Suzuki Y, Yamamoto M, Wada H, Ito M, Nakano T, Sasaki Y, et al Agonist-induced regulation of myosin phosphatase activity in human platelets through activation of Rho-kinase. Blood. 1999;93(10):3408–17. . 10233893

[35] Pleines I, Hagedorn I, Gupta S, May F, Chakarova L, van Hengel J, et al Megakaryocyte-specific RhoA deficiency causes macrothrombocytopenia and defective platelet activation in hemostasis and thrombosis. Blood. 2012;119(4):1054–63. 10.1182/blood-2011-08-372193 . 22045984

[36] Nishioka H, Horiuchi H, Tabuchi A, Yoshioka A, Shirakawa R, Kita T. Small GTPase Rho regulates thrombin-induced platelet aggregation. Biochemical and biophysical research communications. 2001;280(4):970–5. 10.1006/bbrc.2001.4237 . 11162620

[37] Bodie SL, Ford I, Greaves M, Nixon GF. Thrombin-induced activation of RhoA in platelet shape change. Biochemical and biophysical research communications. 2001;287(1):71–6. 10.1006/bbrc.2001.5547 . 11549255

[38] Gao Y, Dickerson JB, Guo F, Zheng J, Zheng Y. Rational design and characterization of a Rac GTPase-specific small molecule inhibitor. Proceedings of the National Academy of Sciences of the United States of America. 2004;101(20):7618–23. 10.1073/pnas.0307512101 15128949 PMC419655

[39] Groemping Y, Rittinger K. Activation and assembly of the NADPH oxidase: a structural perspective. The Biochemical journal. 2005;386(Pt 3):401–16. 10.1042/BJ20041835 15588255 PMC1134858

[40] Diebold BA, Bokoch GM. Molecular basis for Rac2 regulation of phagocyte NADPH oxidase. Nature immunology. 2001;2(3):211–5. 10.1038/85259 . 11224519

[41] Lapouge K, Smith SJ, Walker PA, Gamblin SJ, Smerdon SJ, Rittinger K. Structure of the TPR domain of p67phox in complex with Rac.GTP. Molecular cell. 2000;6(4):899–907. . 11090627 10.1016/s1097-2765(05)00091-2

[42] Handin RI, Karabin R, Boxer GJ. Enhancement of platelet function by superoxide anion. The Journal of clinical investigation. 1977;59(5):959–65. 10.1172/JCI108718 192766 PMC372304

[43] Marcus AJ, Silk ST, Safier LB, Ullman HL. Superoxide production and reducing activity in human platelets. The Journal of clinical investigation. 1977;59(1):149–58. 10.1172/JCI108613 187622 PMC333342

[44] Seno T, Inoue N, Gao D, Okuda M, Sumi Y, Matsui K, et al Involvement of NADH/NADPH oxidase in human platelet ROS production. Thrombosis research. 2001;103(5):399–409. . 11553372 10.1016/s0049-3848(01)00341-3

[45] Chlopicki S, Olszanecki R, Janiszewski M, Laurindo FR, Panz T, Miedzobrodzki J. Functional role of NADPH oxidase in activation of platelets. Antioxidants & redox signaling. 2004;6(4):691–8. 10.1089/1523086041361640 . 15242549

[46] Begonja AJ, Teichmann L, Geiger J, Gambaryan S, Walter U. Platelet regulation by NO/cGMP signaling and NAD(P)H oxidase-generated ROS. Blood cells, molecules & diseases. 2006;36(2):166–70. 10.1016/j.bcmd.2005.12.028 . 16469512

[47] Pignatelli P, Carnevale R, Di Santo S, Bartimoccia S, Sanguigni V, Lenti L, et al Inherited human gp91phox deficiency is associated with impaired isoprostane formation and platelet dysfunction. Arteriosclerosis, thrombosis, and vascular biology. 2011;31(2):423–34. 10.1161/ATVBAHA.110.217885 . 21071703

[48] Getz TM, Dangelmaier CA, Jin J, Daniel JL, Kunapuli SP. Differential phosphorylation of myosin light chain (Thr)18 and (Ser)19 and functional implications in platelets. Journal of thrombosis and haemostasis: JTH. 2010;8(10):2283–93. 10.1111/j.1538-7836.2010.04000.x 20670370 PMC2965805

[49] Leng L, Kashiwagi H, Ren XD, Shattil SJ. RhoA and the function of platelet integrin alphaIIbbeta3. Blood. 1998;91(11):4206–15. . 9596668

[50] Gao G, Chen L, Dong B, Gu H, Dong H, Pan Y, et al RhoA effector mDia1 is required for PI 3-kinase-dependent actin remodeling and spreading by thrombin in platelets. Biochemical and biophysical research communications. 2009;385(3):439–44. 10.1016/j.bbrc.2009.05.090 . 19470376

[51] Gong H, Shen B, Flevaris P, Chow C, Lam SC, Voyno-Yasenetskaya TA, et al G protein subunit Galpha13 binds to integrin alphaIIbbeta3 and mediates integrin "outside-in" signaling. Science. 2010;327(5963):340–3. 10.1126/science.1174779 20075254 PMC2842917

[52] Jirouskova M, Jaiswal JK, Coller BS. Ligand density dramatically affects integrin alpha IIb beta 3-mediated platelet signaling and spreading. Blood. 2007;109(12):5260–9. 10.1182/blood-2006-10-054015 17332246 PMC1890822

