# Supplementary figures and images for: RhoA and Rac1 GTPases Differentially Regulate Agonist-Receptor Mediated Reactive Oxygen Species Generation in Platelets
Source: PLoS One. 2016 Sep 28;11(9):e0163227. doi: 10.1371/journal.pone.0163227 (PMC5040254; doi:10.1371/journal.pone.0163227)

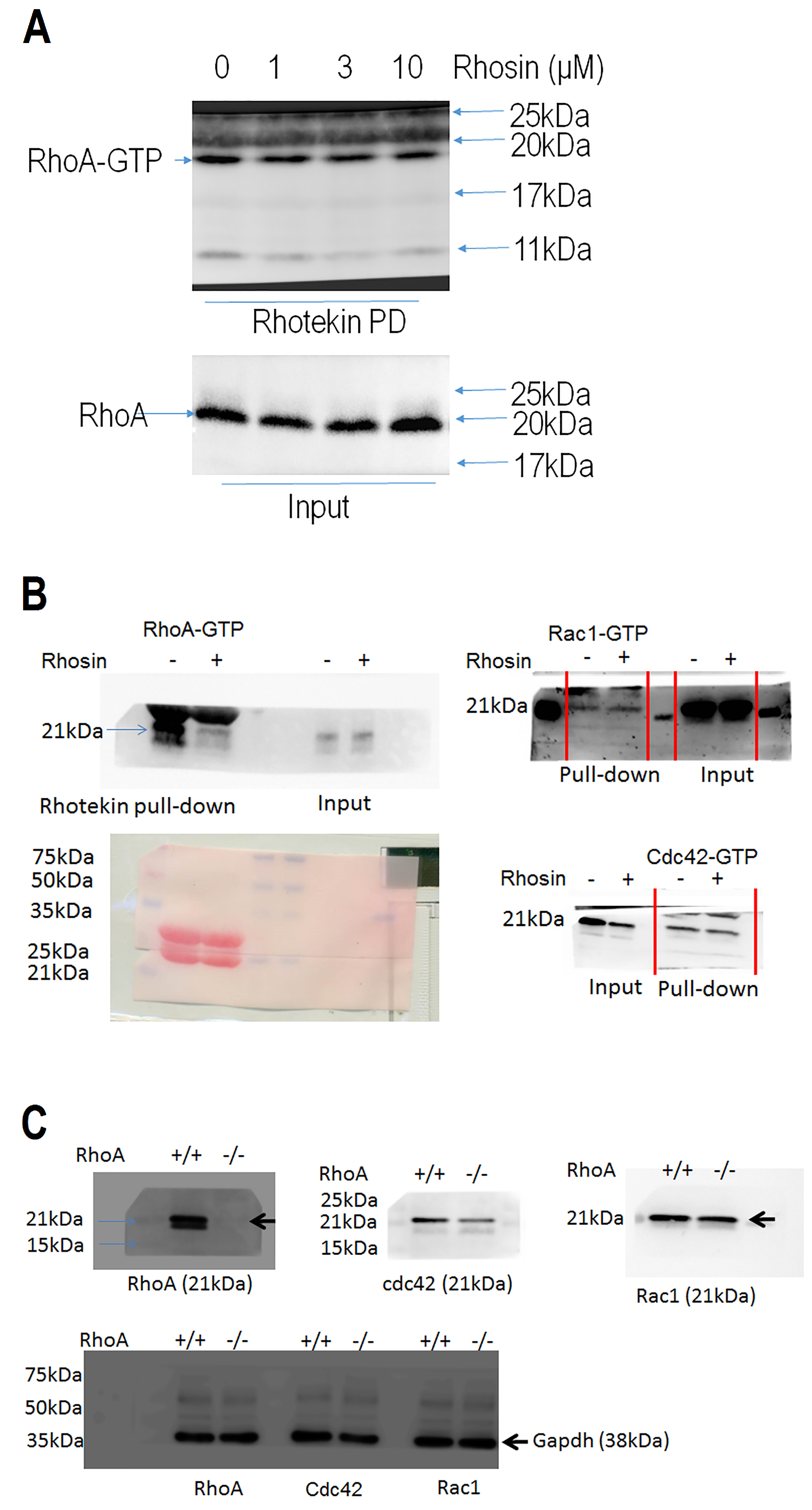

Supplement: S1 Fig — (TIF) [file pone.0163227.s001.tif]

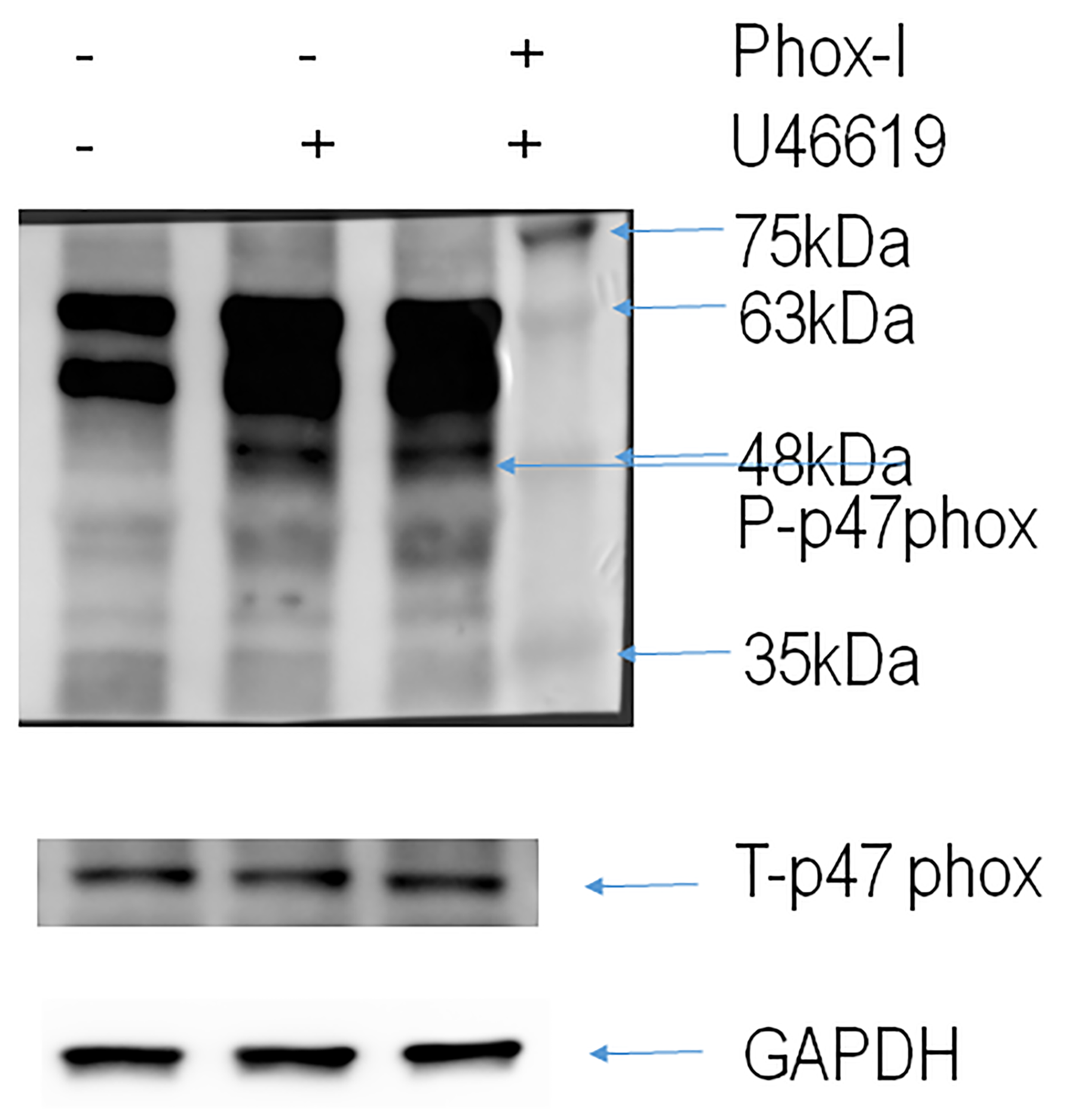

Supplement: S2 Fig — (TIF) [file pone.0163227.s002.tif]

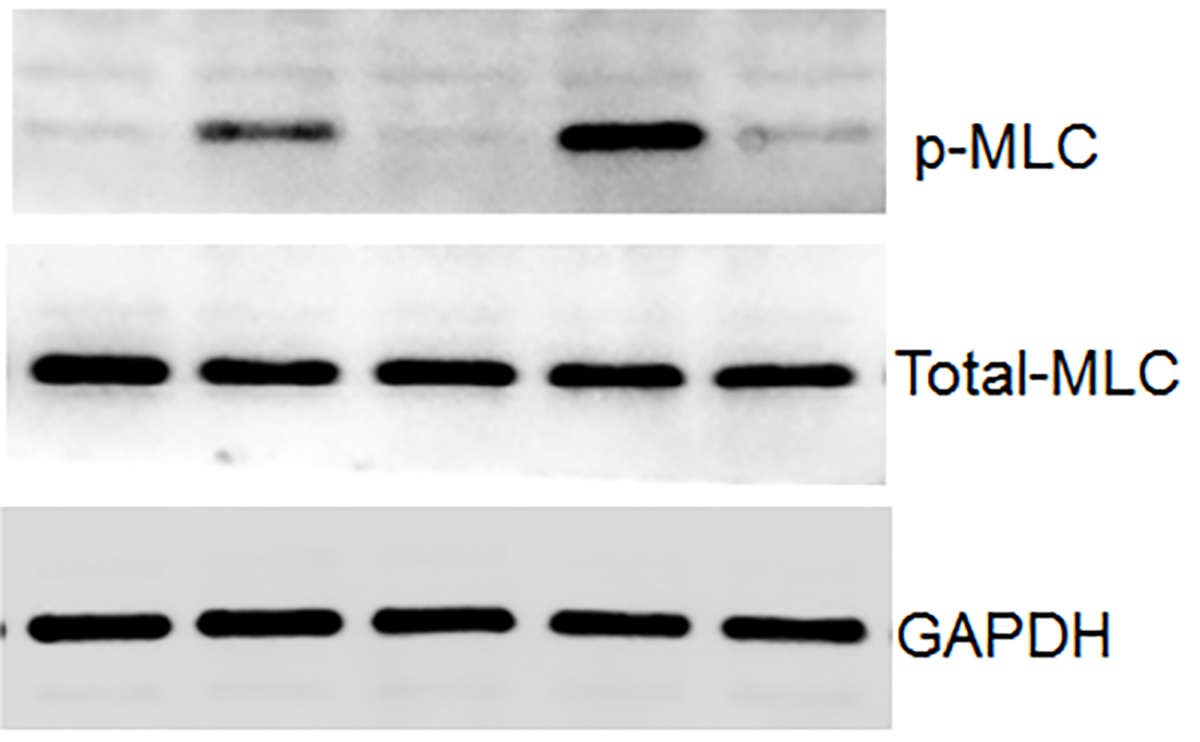

Supplement: S3 Fig — (TIF) [file pone.0163227.s003.tif]

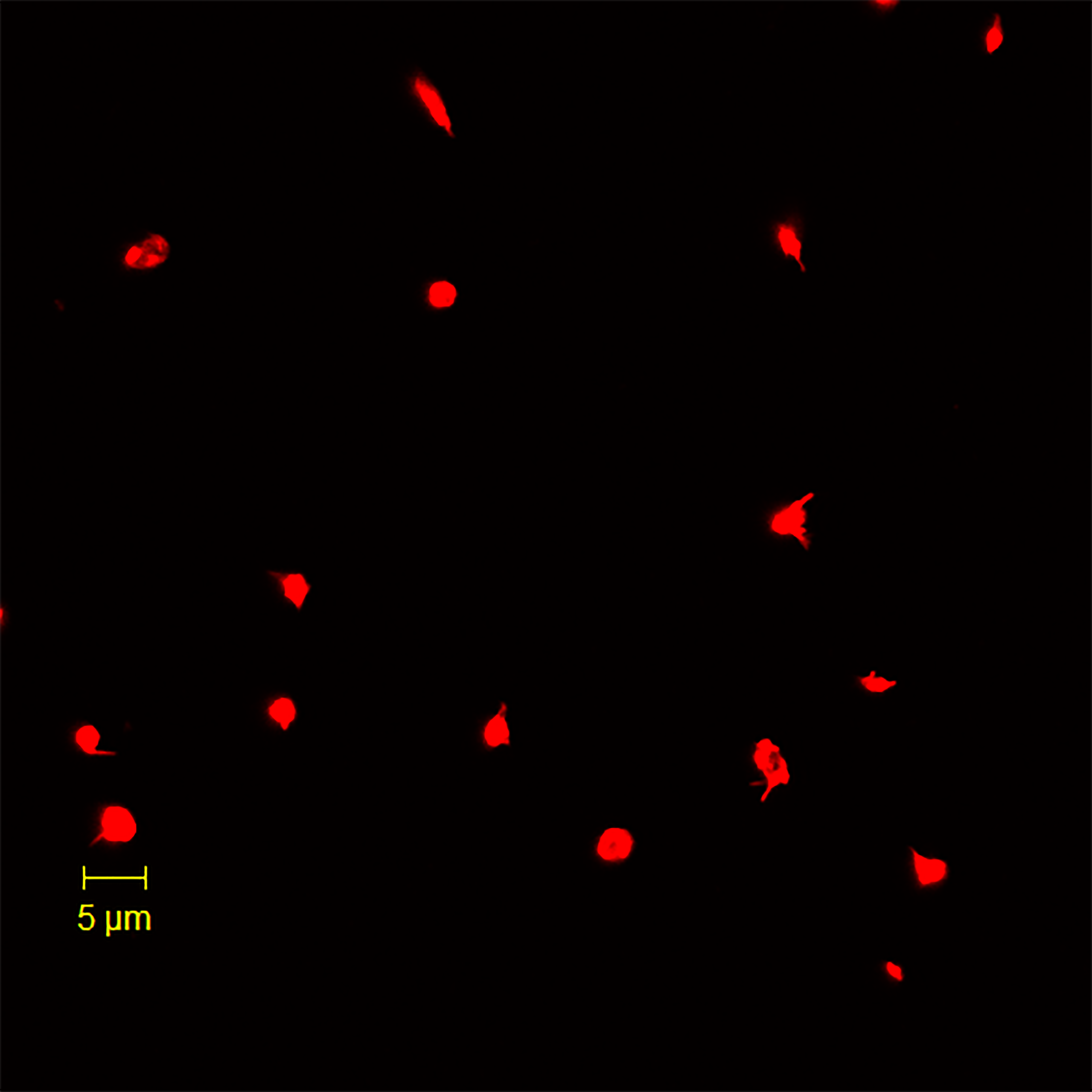

Supplement: S4 Fig — (TIF) [file pone.0163227.s004.tif]
